# The serotonin receptor Am*5HTR2a* links neuromodulation to MRJP1 expression and temporal polyethism in honey bees

**DOI:** 10.3389/fphys.2026.1926478

**Published:** 2026-09-09

**Authors:** Qiushi Wang, Ahmed A. M. Mohamed, Rasha K. Al-Akeel, Hend M. Alharbi, Azza Elgendy, Amr Mohamed, Makio Takeda

**Affiliations:** 1 Graduate School of Agricultural Science, Kobe University, Kobe, Japan; 2 Department of Zoology, Faculty of Science, King Saud University, Riyadh, Saudi Arabia; 3 Department of Biology, College of Science, Princess Nourah Bint Abdulrahman University, Riyadh, Saudi Arabia; 4 Department of Biotechnology, Faculty of Science, Cairo University, Giza, Egypt; 5 Department of Entomology, Faculty of Science, Cairo University, Giza, Egypt

**Keywords:** Am*5HTR2a*, *Apis mellifera*, hypopharyngeal glands, MRJP1, serotonin signaling, temporal polyethism

## Abstract

Division of labor in honey bees is accompanied by coordinated changes in neural and glandular physiology, yet the molecular links between neuromodulatory signaling and Major Royal Jelly Protein 1 (MRJP1) expression remain poorly understood. Here we show that the serotonin receptor Am*5HTR2a* is dynamically associated with nurse-bee physiology and MRJP1 expression in *Apis mellifera*. Among four serotonin receptor subtypes examined, Am*5HTR2a* showed the strongest age-dependent expression pattern. Expression peaked in the brain during the nurse stage (day 11) and slightly later in the hypopharyngeal glands (HG; day 16), where it rose from undetectable levels to a peak relative expression level of approximately 98, normalized to *rp49*, whereas the other receptor transcripts remained comparatively stable. Pharmacological manipulation showed that serotonin increased Am*5HTR2a* and *MRJP1* expression in the brain and HG in a dose-dependent manner, whereas melatonin produced the opposite effect. A multifactorial general linear model identified pharmacological treatment as the dominant predictor of Am*5HTR2a* expression (*F*_2_,_22_ = 65.31, *p* < 0.001, partial η² = 0.856; *n* = 3 biological replicates per group), with a significant tissue × treatment interaction (*F*_2_,_22_ = 7.58, *p* = 0.003, partial η² = 0.408), indicating that serotonergic and melatonergic effects differed between neural and exocrine tissues. Consistently, endogenous serotonin depletion with 5,7-dihydroxytryptamine reduced *MRJP1* expression. RNA interference-mediated knockdown of Am*5HTR2a* efficiently suppressed receptor transcript abundance and was accompanied by a progressive decline in MRJP1 mRNA. Notably, serotonin remained able to elevate *MRJP1* expression in forager bees in a dose-dependent manner, indicating that serotonergic responsiveness persists beyond the nurse stage and that aspects of temporal polyethism remain physiologically reversible. Together, these results identify Am*5HTR2a* as a component of a serotonergic signaling pathway associated with worker bee physiology and HG-associated *MRJP1* expression. The findings support a model in which serotonergic and melatonergic signaling are potentially linked to arylalkylamine *N*-acetyltransferase (AANAT)-dependent indoleamine metabolism, although AANAT activity was not directly measured here. In this framework, these signals help shape temporal polyethism by modulating molecular programs underlying worker allocation to behavioral and metabolic tasks.

## 1 Introduction

Division of labor is a defining feature of eusocial insects and is particularly well developed in the Western honey bee, *Apis mellifera*. Worker bees undergo age-related behavioral transitions known as temporal polyethism, in which young adults typically perform in-hive tasks such as brood care and food processing before shifting to foraging later in life (Johnson, 2008; Schilcher and Scheiner, 2023). This transition is highly plastic and reflects the integration of colony demands, nutritional state, endocrine signaling, and environmental inputs acting across multiple physiological systems (Ament et al., 2008; Hamilton et al., 2019; Fang et al., 2023). Accompanying these behavioral shifts are extensive physiological changes, including remodeling of the hypopharyngeal glands (HG), alterations in metabolism, and changes in neuromodulatory signaling pathways that collectively support task specialization within the colony (Hamilton et al., 2019; Schilcher and Scheiner, 2023).

The HG are central to nurse bee physiology because they synthesize and secrete royal jelly, the protein-rich brood food required for larval nutrition and queen development. During the nurse stage, the glands exhibit high secretory activity and elevated production of major royal jelly proteins (MRJPs), whereas glandular activity declines substantially as workers transition to foraging (Knecht and Kaatz, 1990; Corona et al., 2023). Among the MRJPs, MRJP1, historically referred to as “royalactin” (RA) following Kamakura’s original hypothesis, has attracted particular attention following reports that it contributes to queen-associated developmental traits in honey bee larvae (Kamakura, 2011; Fang et al., 2023). Royalactin, a monomeric form of MRJP1, has been reported to exert EGF-like proliferative effects (Kamakura, 2002) and, in *Drosophila*, to increase body size, fecundity, and lifespan while shortening developmental time through EGFR-related signaling pathways (Kamakura, 2011). Subsequent work, however, has emphasized that caste differentiation cannot be attributed to a single factor and instead emerges from interactions among nutrition, endocrine regulation, metabolism, and epigenetic mechanisms (Maleszka, 2018; Alhosin, 2023). While the role of MRJP1 in caste determination remains controversial, its expression is strongly associated with HG function and royal jelly production in nurse honey bees, reflecting its participation in a broader nutrigenomic and endocrine regulatory network (Kamakura, 2011; Maleszka, 2018; Peng et al., 2024; Hassanyar et al., 2025; Wang et al., 2025; Fang et al., 2026).

Behavioral maturation in worker honey bees is coordinated by multiple endocrine and neuromodulatory systems. Juvenile hormone (JH) is strongly associated with the transition from nursing to foraging, while vitellogenin and nutritional signaling pathways are linked to brood-care physiology and metabolic state (Shalem et al., 2025; Unnikrishnan et al., 2026). Vitellogenin is an established regulator of honey bee physiological maturation and division of labor and interacts with juvenile hormone, nutritional state, and octopamine signaling (Schilcher and Scheiner, 2023). Biogenic amines also play important roles in regulating sensory responsiveness, locomotion, learning, feeding behavior, and task allocation in honey bees and other insects (Schulz and Robinson, 1999; Buckemüller et al., 2017; Linn et al., 2020; Barbero and Casacci, 2025). Among these neuromodulators, serotonin (5-hydroxytryptamine; 5-HT) has emerged as a candidate regulator of behavioral and physiological plasticity because of its widespread functions in both the central nervous system and peripheral tissues (Thamm et al., 2013; Tedjakumala et al., 2014; Raza and Li, 2025). Together, these interconnected endocrine, nutritional, and neuromodulatory pathways form a complex regulatory network underlying worker behavioral and physiological transitions. Recent advances in honey bee genome editing are beginning to provide more precise functional approaches for investigating such pathways: an *in silico* CRISPR/Cas9 framework has been developed for the *A. mellifera* vitellogenin gene (Davoodi et al., 2026), while a liposome-mediated CRISPR/Cas9 approach using drone sperm has achieved heritable editing of the juvenile hormone acid methyltransferase gene (Yıldız and Karabağ, 2026).

Honey bees (*Apis mellifera*) possess four molecularly characterized serotonin receptor subtypes: Am5HTR1, Am5HTR2a, Am5HTR2b, and Am5HTR7 (Thamm et al., 2010, 2013), with receptor nomenclature updated here according to current NCBI GenBank/RefSeq records. These receptors belong to the evolutionarily conserved 5-HT_1_, 5-HT_2_, and 5-HT_7_ receptor families found across insects and vertebrates (Thamm et al., 2010; Bacqué-Cazenave et al., 2020). Previous studies have demonstrated that *Am*5HTR2 receptors are broadly expressed in the honey bee brain and in exocrine tissues, including the HG, where transcript levels are particularly high, suggesting a potential role in integrating neural activity with peripheral secretory functions (Thamm et al., 2013). Serotonergic signaling has additionally been implicated in feeding-related physiology, gut motility, and behavioral responsiveness in honey bees (French et al., 2014). In contrast, melatonin, biosynthesized from serotonin via the arylalkylamine metabolic pathway, is involved in circadian regulation and behavioral state transitions in insects, although its functional interaction with serotonergic signaling in honey bee temporal polyethism remains incompletely resolved (Yang et al., 2007; Izawa et al., 2023; Nahar et al., 2026).

In our previous studies, serotonin–melatonin balance was proposed to function as a developmental switch in the oak silkmoth, *Antheraea pernyi* (Wang et al., 2013; Mohamed et al., 2014). Moreover, exogenous melatonin was shown to induce nurse-to-forager behavioral transition in honey bees (Nahar et al., 2026), suggesting a potential antagonistic relationship between serotonergic and melatonergic signaling during worker maturation. Building on this framework, we hypothesized that serotonin signaling is involved in sustaining nurse-stage physiology, including royal jelly production, in contrast to the foraging-promoting action of melatonin.

Although the endocrine regulation of worker behavioral maturation has been extensively investigated, the molecular pathways linking neuromodulatory signaling to HG-associated royal jelly production remain poorly resolved. In particular, it remains unclear whether serotonergic signaling contributes directly to the regulation of MRJP1 expression during the nurse-to-forager transition. The present study addressed this question by examining age-dependent expression patterns of honey bee serotonin receptor genes in the brain and HG, with particular focus on Am*5HTR2a*. Using pharmacological manipulation, RNA interference (RNAi), serotonergic depletion, and multifactorial statistical modeling, we investigated the relationship between serotonergic signaling and MRJP1 expression across worker developmental stages. Our results identify Am5HTR2a as a receptor subtype strongly associated with nurse-stage physiology and support a role for serotonergic signaling in regulating molecular features linked to HG activity and royal jelly production.

## 2 Materials and methods

### 2.1 Insects

Honey bees (*A. mellifera*), Italian golden strain, used for the analyses were collected from colonies maintained in the experimental garden of Kobe University, Kobe, Hyogo, Japan. The source colony was originally purchased from Mamuro Apiary (Japan) and maintained under standard apicultural conditions (Ohtani, 1974; Izawa et al., 2023). Newly emerged adults, distinguished morphologically from older workers, were collected from the main hive, transferred to an observation hive, and individually marked with numbered paper tags. Bees were dissected in phosphate-buffered saline (PBS), and the brain and HG were collected. Dissected tissues were stored at −80 °C until mRNA quantification. The observation hive contained a queen sibling of the queen from the main hive and approximately 20 nurse bees. Bees sampled from the field hive were transferred to the observation hive within 1 day after marking. The observation hive was maintained in a semi-indoor space at approximately 30 °C using a heater. Experimental procedures for colony maintenance, worker marking, tissue dissection, dsRNA preparation, hemocoelic injection, and RT-qPCR were performed according to our previously validated protocols (Mohamed et al., 2014; Nahar et al., 2026), with assay-specific modifications described below.

Chronological age (Day 11 and Day 21) was assigned from the marked date of adult emergence and was used throughout this study to represent nurse- and forager-stage workers, respectively. Because temporal polyethism is plastic, chronological age is only an approximate predictor of individual behavioral state; residual variation in task performance within each age group cannot be excluded. All bees were derived from a single source colony, with the observation hive headed by a queen sibling of the main-hive queen. Consequently, the present dataset does not capture colony-to-colony genetic or environmental variation, which may limit the broader generalizability of the findings. Both limitations are considered further in the Discussion (Section 4).

### 2.2 Isolation of total RNA and cDNA synthesis

Total RNA was isolated from dissected tissues using RNAiso Plus reagent (Takara, Japan) according to the manufacturer’s instructions. RNA (1 µg) was reverse-transcribed in 7 µL nuclease-free water and 2 µL 5× RT Master Mix using the ReverTra Ace kit (Toyobo Co. Ltd., Osaka, Japan). Reverse transcription was performed at 65 °C for 5 min, 37 °C for 15 min, 50 °C for 5 min, and 98 °C for 5 min, followed by incubation at 4 °C.

### 2.3 Preparation and injection of dsRNA

A 202-bp fragment of Am*5HTR2a* (accession number NM_001202460.1; last accessed 15 May 2026) was amplified using gene-specific primers (Am*5HTR2a*-T7-F and Am*5HTR2a*-T7-R) (**Supplementary Table 1**), each carrying a T7 promoter sequence at the 5′ end. The purified PCR product was incubated with the MEGAscript RNAi kit (Ambion, CA, USA) at 37 °C for 4 h according to the manufacturer’s instructions, and double-stranded RNA (dsRNA) was synthesized. Control dsRNA was generated from the jellyfish GFP gene (dsRNA-GFP), which was used as a negative control and was not expected to affect the target gene (Tschuch et al., 2008). Before injection, dsRNA and Metafectene PRO (Biontex, Planegg, Germany) were mixed at a 1:1 (v:v) ratio. Each bee received 750 ng dsRNA. Because *Am5HTR2a* and *Am5HTR2b* share substantial sequence homology, the 202-bp dsRNA was designed to target a region of *Am5HTR2a* with minimal predicted complementarity to *Am5HTR2b*. The *Am5HTR2a* RT-qPCR primers were positioned outside the dsRNA target region to avoid amplification of the introduced dsRNA. The target region was selected to minimize potential off-target effects, although *Am5HTR2b* and other receptor transcripts were not assessed following RNAi treatment.

### 2.4 Quantitative real-time PCR

Quantitative real-time PCR (RT-qPCR) was performed using SYBR^®^ Green and THUNDERBIRD™ qPCR Mix (Toyobo Co. Ltd., Osaka, Japan). Forward and reverse primers are listed in **Supplementary Table 1**. Amplification specificity was confirmed by melting-curve analysis using ABI 7000 Sequence Detection System software (Applied Biosystems, Foster City, CA, USA). Each reaction contained 2.5 µL cDNA and 22.5 µL PCR master mix containing mix, nuclease-free water, primers, and ROX dye. Cycling conditions were 95 °C for 1 min, followed by 40 cycles of 95 °C for 15 s and 60 °C for 1 min. Relative transcript abundance was normalized to *rp49* mRNA (accession number AF441189), which served as the internal reference gene, and relative expression calculated using the 2^−ΔΔCt^ method (Livak and Schmittgen, 2001). For Am*5HTR2a*, the primers used for RT-qPCR (**Supplementary Table 1**) were designed outside the dsRNA target region to avoid amplification of the knockdown fragment. The expected amplicon sizes were 222 bp for Am*5HTR1*, 209 bp for Am*5HTR2a*, 212 bp for Am*5HTR2b*, and 208 bp for Am*5HTR7*. The *rp49* transcript was used as the sole reference gene for normalization throughout the study, consistent with its established use in honey bee gene expression studies (Lourenço et al., 2012; Bomtorin et al., 2014; Moon et al., 2018; Jeon et al., 2020). The suitability of *rp49* under the specific conditions examined here was not specifically assessed.

### 2.5 PCR

To examine the effect of pharmacological and RNAi treatments on *MRJP1* expression, bees of known age were injected using a microsyringe (Hamilton 10 µL). Relative *MRJP1* mRNA levels were quantified by RT-qPCR using the primers listed in **Supplementary Table 1**, following the same protocol described in Section 2.4, with expression normalized to *rp49* and relative abundance calculated using the 2^−ΔΔCt^ method. In addition, conventional PCR was performed using the KOD FX kit (Toyobo Co. Ltd., Japan; KFX-101) according to the manufacturer’s instructions to verify amplification specificity and visualize *MRJP1* expression patterns; PCR products were separated on a 1% agarose gel.

### 2.6 Injection of 5-HT, melatonin, and 5,7-dihydroxytryptamine

A total of 0.1, 1, or 10 pmol 5-HT in 5 µL distilled water, or an equimolar amount of melatonin in 5 µL ethanol, was injected into each bee using a microsyringe. Similarly, 0.1, 1, or 10 pmol 5,7-DHT (Sigma, USA) in 5 µL distilled water was injected into each bee using a microsyringe. Bees injected with the same volume of distilled water or ethanol served as controls. Bees were dissected 24 h after injection, total RNA was extracted from the brain and HG, and gene expression was analyzed by PCR. The 0.1, 1, and 10 pmol doses were selected as a graded pharmacological series based on dose ranges used in our previous biogenic-amine injection studies (Mohamed et al., 2014; Nahar et al., 2026). Endogenous concentrations were not measured in the present study.

### 2.7 Statistical analysis

All statistical analyses were conducted in R version 4.5.2 (R Core Team, 2025). Data are presented as mean ± standard error of the mean (SEM) from three independent biological replicates (n = 3). Prior to inferential testing, model residuals were assessed for normality using the Shapiro–Wilk test and for homogeneity of variance using Levene’s test. Where parametric assumptions were met, data were analyzed using analysis of variance (ANOVA) or general linear models (GLMs), as appropriate. For the age-dependent expression analysis of Am*5HTR2a* in the HG, where assumptions of normality and homogeneity of variance were not satisfied, the Kruskal–Wallis test was used. For all omnibus tests showing significant effects, *post hoc* pairwise comparisons were performed using Tukey’s honestly significant difference (HSD) test with familywise error rate control at α = 0.05. For the Kruskal–Wallis analysis, pairwise comparisons were adjusted for multiple testing using Dunn’s test with Holm correction. Statistical significance was set at α = 0.05 for all analyses.

For all experiments, n = 3 represents three independent biological replicates based on separately collected tissue samples. Technical replicates, when performed, were repeated measurements of the same biological sample and were not treated as independent observations. The relatively small number of biological replicates may limit statistical power and is acknowledged as a limitation in the Discussion (Section 4).

#### 2.7.1 Ontogenetic expression of serotonin receptors in the brain and hypopharyngeal glands

To profile developmental expression patterns, relative mRNA levels of the four receptor subtypes (Am*5HTR1*, Am*5HTR7*, Am*5HTR2a*, and Am*5HTR2b*) in the brain and Am*5HTR2a* expression in the HG were measured across six ontogenetic time points: days 3, 6, 11, 16, 21, and 23. Differences in brain receptor expression were analyzed using a two-way ANOVA with gene (Am*5HTR1*, Am*5HTR2a*, Am*5HTR2b*, and Am*5HTR7*) and worker age (days 3, 6, 11, 16, 21, and 23) as fixed factors, because these data satisfied parametric assumptions. This design allowed detection of a Gene × Day interaction, which was used to determine whether receptor subtypes differed in their age-dependent expression trajectories. *Post-hoc* pairwise comparisons were performed using Tukey’s HSD test. Relative Am*5HTR2a* expression in the HG was analyzed using the Kruskal–Wallis test, followed by pairwise *post hoc* comparisons as noted above.

#### 2.7.2 Multifactorial general linear modeling of Am*5HTR2a* expression

A GLM was fitted to evaluate relative Am*5HTR2a* expression in response to three fixed factors: tissue type (brain vs. HG), worker age (day 11 vs. day 21), and pharmacological treatment (control, 5-HT, and melatonin). Because melatonin was administered only to day 11 bees, the Age × Treatment and Tissue × Age × Treatment terms were non-estimable and were excluded from the final model. The resulting GLM included the main effects of tissue, age, and treatment, together with the estimable two-way interactions Tissue × Age and Tissue × Treatment. To accommodate the unbalanced design, Type III (marginal) sums of squares were calculated using the car package in R. *Post hoc* pairwise comparisons among treatment means were conducted using Tukey-adjusted estimated marginal means via the *emmeans* package. Effect sizes were calculated as partial eta squared (ηp²) using the *effectsize* package and interpreted according to standard benchmarks: small (ηp² ≥ 0.01), medium (ηp² ≥ 0.06), and large (ηp² ≥ 0.14).

#### 2.7.3 Temporal dynamics of dsRNA-mediated Am*5HTR2a* knockdown and MRJP1 expression

To validate transcriptional knockdown efficiency and assess downstream physiological effects, 11-day-old bees were injected with dsRNA (dsRNA-Am5HTR2a, dsRNA-GFP control, or injection-free control) and sampled at 0, 24, 48, and 72 h post-injection. Knockdown efficiency was evaluated using a two-way ANOVA with treatment, time, and their interaction as fixed effects on relative Am*5HTR2a* mRNA levels. *Post hoc* pairwise comparisons between treatment groups at each time point were performed using Tukey’s HSD test. To evaluate the downstream consequences of receptor knockdown, relative *MRJP1* expression over the same post-injection time course (0, 24, 48, and 72 h) was analyzed using one-way ANOVA, with time post-injection as the sole fixed factor.

#### 2.7.4 Pharmacological modulation of MRJP1 expression

The regulatory effects of serotonergic, melatonergic, and neurotoxic manipulations on *MRJP1* expression were investigated in the brain and hypopharyngeal glands (HG) of 11- and 21-day-old worker bees using a series of two-way ANOVAs. First, a two-way ANOVA was used to assess the effects of tissue (brain vs. HG), pharmacological treatment (control, vehicle, and 0.1, 1, or 10 pmol of either 5-HT or melatonin), and their interaction on relative *MRJP1* expression. *Post hoc* multiple comparisons were conducted using Tukey’s HSD test, with estimated marginal means calculated for treatment within each tissue. Second, a separate two-way ANOVA evaluated the interaction between tissue and 5-HT dose as the primary factors to resolve dose-dependent regulatory patterns. Third, to investigate serotonergic depletion, a two-way ANOVA was performed to evaluate the main effects and interaction of tissue (brain vs. HG) and treatment (control, vehicle, and 5,7-DHT at 0.1, 1, or 10 pmol) on relative *MRJP1* levels. For all pharmacological experiments, pairwise differences among treatment groups within each tissue were evaluated using Tukey’s honestly significant difference (HSD) test implemented through the emmeans package in R.

## 3 Results

### 3.1 Ontogenetic expression profiles of serotonin receptor genes in the brain and hypopharyngeal glands

**Figure 1:**
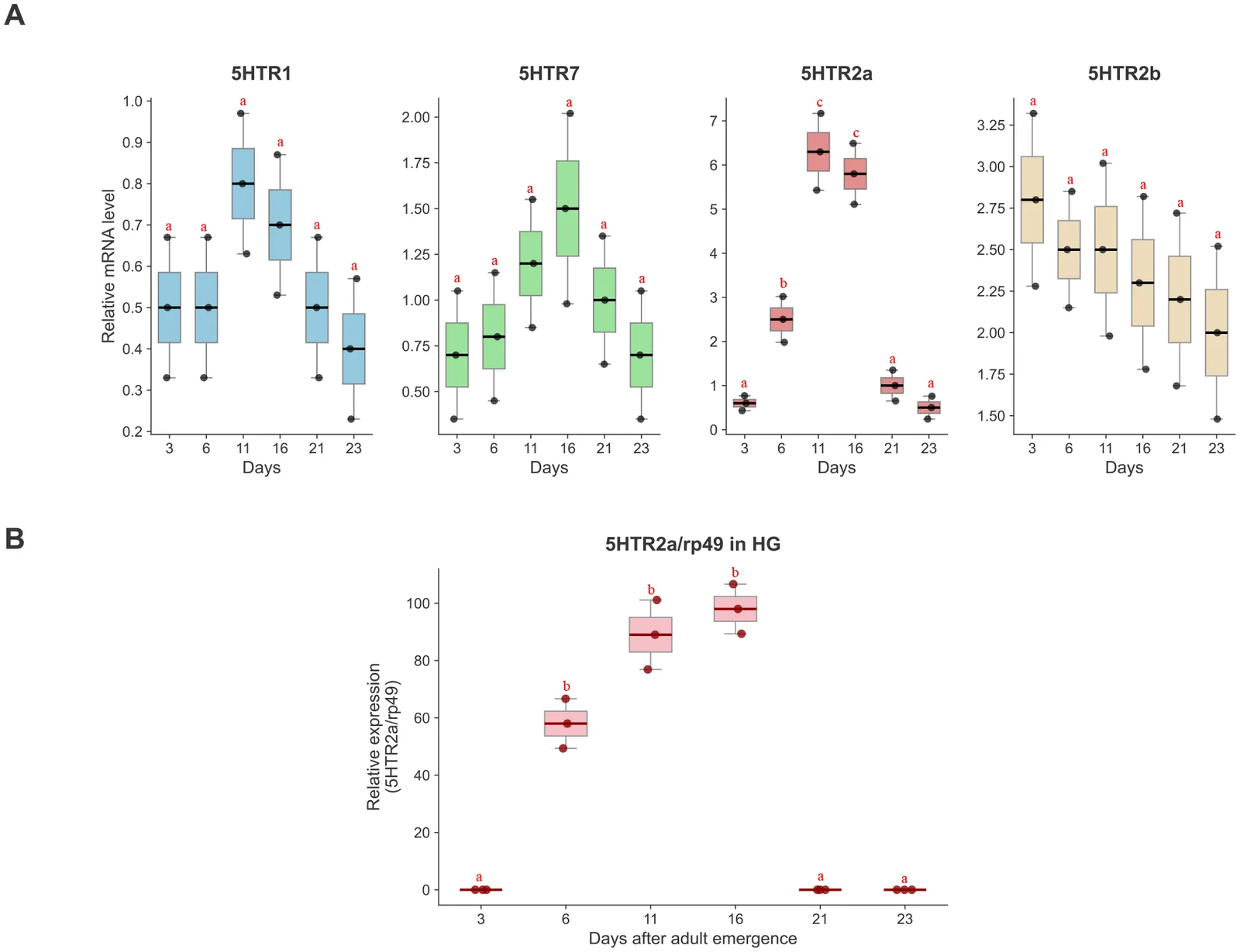
Temporal expression profiles of serotonin receptor genes (*Am5HTRs*) in the honeybee brain and hypopharyngeal glands (HG) following adult emergence. **(A)** Relative mRNA levels of Am*5HTR1*, Am*5HTR7*, Am*5HTR2a*, and Am*5HTR2b* in the brain at days 3, 6, 11, 16, 21, and 23 post-adult emergence, as determined by quantitative real-time PCR (qRT-PCR). Expression levels were normalized to the reference gene *rp49* and are presented as mean ± S.E.M. from three independent biological replicates (n = 3). Statistical differences across time points were assessed by Two-Way ANOVA (Gene × Day interaction) followed by Tukey’s HSD *post-hoc* test; groups sharing the same letter (a, b, or c) are not significantly different from one another (*p* > 0.05). **(B)** Relative expression of Am*5HTR2a* normalized to *rp49* in the hypopharyngeal glands (HG) across the same developmental time points. Statistical differences were determined by the Kruskal-Wallis test, with pairwise comparisons conducted using Dunn’s test with Holm correction; different letters denote statistically significant differences between groups (*p* < 0.05). Box plots display the median (horizontal line), mean (filled circle), interquartile range (box), and 1.5× IQR (whiskers); outliers are shown as individual points.

To establish baseline expression dynamics of the four serotonin receptor subtypes across the adult lifespan of *Apis mellifera* workers, relative mRNA levels of Am*5HTR1*, Am*5HTR7*, Am*5HTR2a*, and Am*5HTR2b* were quantified in the brain at six developmental time points (Days 3, 6, 11, 16, 21, and 23 post-adult emergence) by quantitative real-time PCR (qRT-PCR), normalized to the reference gene *rp49* (**Figure 1A**; **Supplementary Table 2**).

Among the four receptor subtypes examined, Am*5HTR2a* displayed the most pronounced and dynamic age-dependent expression pattern in the brain. Expression of Am*5HTR2a* was low at Day 3 (mean = 0.60 ± 0.10), increased sharply to 2.50 ± 0.30 by Day 6, and peaked at Day 11 (6.30 ± 0.50), coinciding with the nurse bee stage. Expression remained elevated at Day 16 (5.80 ± 0.40) before declining precipitously by Day 21 (1.00 ± 0.20), the age typically associated with the transition to foraging, and reaching a nadir at Day 23 (0.50 ± 0.15). Two-way ANOVA revealed a significant Gene × Day interaction (*p* < 0.05), and Tukey’s HSD *post-hoc* comparisons confirmed that Am*5HTR2a* expression at Days 11 and 16 was significantly higher than at all other time points (*p* < 0.05; **Figure 1A**).

In contrast to the dramatic fluctuations observed for Am*5HTR2a*, the other three receptor subtypes exhibited comparatively stable expression across the adult lifespan. Am*5HTR1* expression remained low and relatively invariant (range: 0.40–0.80), showing a modest, non-significant peak at Day 11. Am*5HTR7* displayed a gradual increase from Day 3 (0.70 ± 0.20) to Day 16 (1.50 ± 0.30), followed by a decline toward Day 23 (0.70 ± 0.20). *Am5HTR2b* showed a slight but consistent downward trend across time points, declining from 2.80 ± 0.30 at Day 3 to 2.00 ± 0.30 at Day 23 (**Supplementary Table 2**).

Given the striking age-dependent modulation of Am*5HTR2a* in the brain, its expression was further characterized in the HG, the primary royal jelly-producing organ in nurse bees. Am*5HTR2a* expression in the HG mirrored the temporal profile observed in the brain but was markedly more pronounced (**Figure 1B**; **Supplementary Table 3**). Expression was undetectable at Day 3, rose substantially by Day 6 (58.00 ± 5.00), peaked at Day 16 (98.00 ± 5.00), and declined to undetectable levels by Day 21. A Kruskal-Wallis test confirmed a significant effect of age on Am*5HTR2a* HG expression (*p* < 0.05), with pairwise *post hoc* comparisons using Dunn’s test with Holm correction revealing significant differences between the peak expression period (Days 11–16) and the early/late time points (Days 3, 21, and 23; *p* < 0.05).

Peak normalized *Am5HTR2a* expression was higher in the HG (~98 at Day 16) than in the brain (~6.3 at Day 11; **Figures 1A, B**), although these independently normalized values from different tissues do not represent absolute transcript abundance.

### 3.2 Multifactorial determinants of Am*5HTR2a* expression

To disentangle the relative contributions of tissue type, worker age, and pharmacological treatment to Am*5HTR2a* expression, a General Linear Model (GLM) was fitted using Type III Sums of Squares (**Table 1**; **Supplementary Table 4**). The GLM revealed that pharmacological treatment was the strongest predictor of Am*5HTR2a* expression, accounting for the largest proportion of variance (*F*_2_,_22_ = 65.310, *p* < 0.001, partial η² = 0.856). This large effect size indicated that treatment explained approximately 85.6% of the variance in Am*5HTR2a* expression after controlling for other factors.

**Table 1:** General linear model (GLM) with type III sums of squares for Am5HTR2a relative mRNA level.

Source of variation	SS (type III)	df	F	p-value	Partial eta squared
Tissue	8.760	1	11.201	0.003 **	**0.337**
Age (days)	9.004	1	11.512	0.003 **	**0.344**
Treatment	102.156	2	65.310	< 0.001 ***	**0.856**
Tissue x Age	0.454	1	0.580	0.454	0.026
Tissue x Treatment	11.856	2	7.580	0.003 **	**0.408**
Residuals (Error)	17.206	22	–	–	–

Model: mRNA ~ Tissue + Age + Treatment + Tissue x Age + Tissue x Treatment. Age x Treatment and three-way interaction terms excluded (Melatonin administered only at Day 11, rendering those terms non-estimable). SS, Sums of Squares; df, degrees of freedom; F, F-statistic.

Bold values indicate statistically significant effects (p < 0.05). **p < 0.001; *p < 0.01.

Tissue type (Brain vs. HG) also exerted a significant main effect (*F*_1_,_22_ = 11.201, *p* = 0.003, partial η² = 0.337), as did worker age (Day 11 vs. Day 21; *F*_1_,_22_ = 11.512, *p* = 0.003, partial η² = 0.344); both represent large effect sizes by conventional benchmarks, indicating that tissue compartment and developmental stage each account for a substantial proportion of variance in Am*5HTR2a* expression independent of pharmacological treatment. Notably, a significant Tissue × Treatment interaction was observed (*F*_2_,_22_ = 7.580, *p* = 0.003, partial η² = 0.408), indicating that the magnitude of treatment effects on Am*5HTR2a* expression differed between the brain and HG. The Tissue × Age interaction was not significant (*F*_1_,_22_ = 0.580, *p* = 0.454, partial η² = 0.026; **Table 1**).

**Figure 2:**
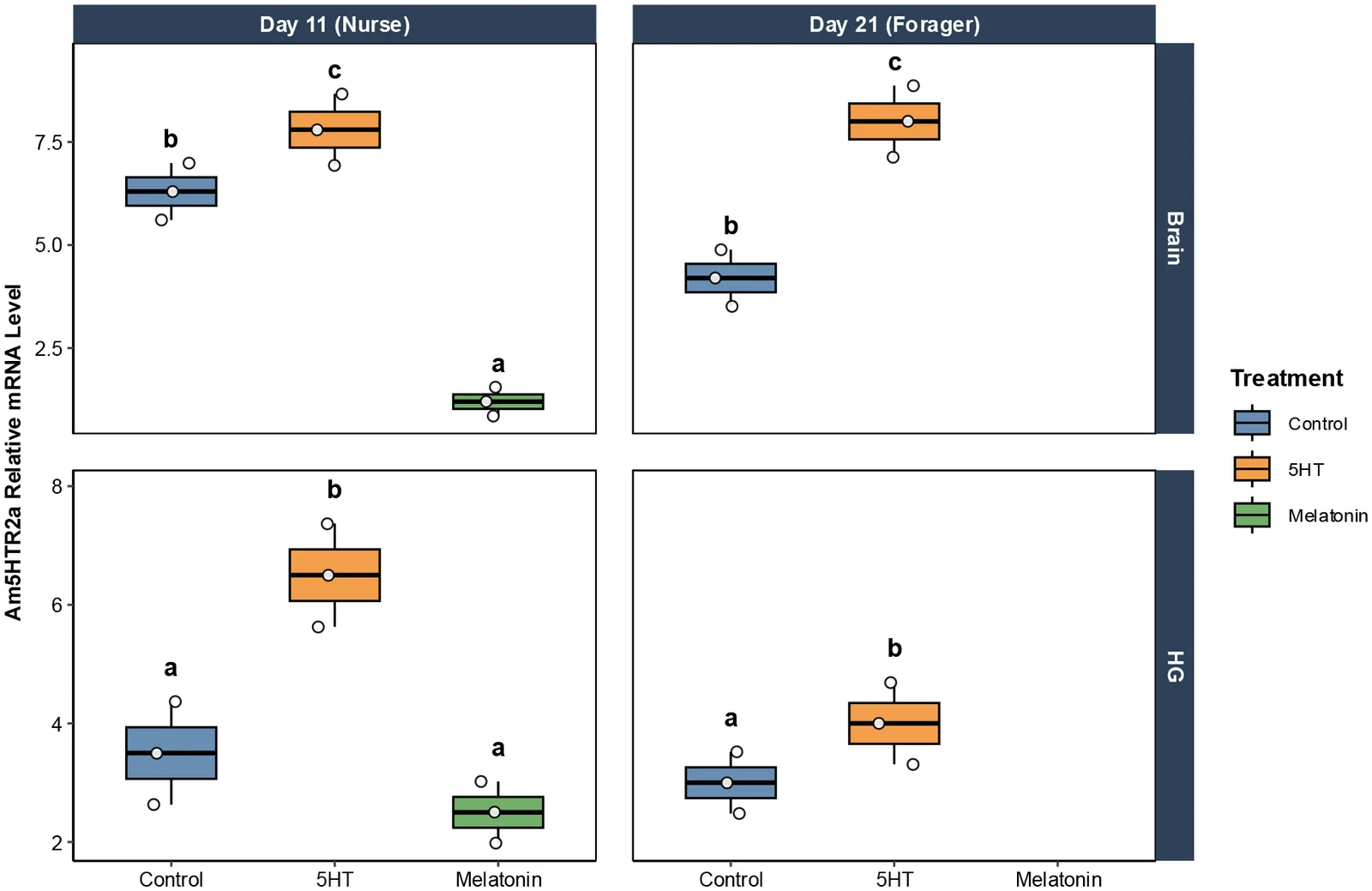
Effects of 5-HT and melatonin injections on Am5HTR2a relative mRNA expression in the brain and HG of *Apis mellifera* workers at two ages. Relative Am*5HTR2a* mRNA levels were measured in nurse bees (Day 11) and forager bees (Day 21) following injection of serotonin (5-HT), melatonin, or vehicle (Control). Boxes represent the median and interquartile range (IQR); whiskers extend to 1.5 × IQR; open circles denote individual biological replicates (n = 3 per group). Different letters above boxes indicate statistically significant differences among treatment groups within each tissue–age combination, as determined by a General Linear Model (GLM) with Type III Sums of Squares followed by Tukey’s Honestly Significant Difference (HSD) *post-hoc* test adjusted for multiple comparisons (familywise α = 0.05).

*Post-hoc* analysis of the GLM results, corroborated by direct experimental comparison, revealed tissue- and age-specific responses of Am*5HTR2a* to serotonergic and melatonergic modulation (**Figure 2**; **Supplementary Table 4**). In nurse bee brains (Day 11), 5-HT injection significantly upregulated Am*5HTR2a* expression (7.80 ± 0.87) compared to control bees (6.30 ± 0.69), whereas melatonin treatment dramatically suppressed expression to 1.20 ± 0.35, representing an approximate 81% reduction relative to control levels (*p* < 0.05; Tukey’s HSD). In forager bee brains (Day 21), 5-HT injection elicited a marked upregulation of Am*5HTR2a* (8.00 ± 0.87) compared to age-matched controls (4.20 ± 0.69; *p* < 0.05).

In the HG, a similar directional pattern was observed. 5-HT significantly increased Am*5HTR2a* expression in both nurse bees (6.50 ± 0.87 vs. 3.50 ± 0.87 in controls) and forager bees (4.00 ± 0.69 vs. 3.00 ± 0.52 in controls), while melatonin suppressed HG expression in nurse bees (2.50 ± 0.52 vs. 3.50 ± 0.87 in controls). These tissue-specific treatment responses are consistent with the significant Tissue × Treatment interaction identified in the GLM (**Table 1**), underscoring that the serotonergic regulatory axis differentially modulates Am*5HTR2a* transcription in neuroendocrine versus exocrine tissues (**Figure 2**). The effects of 5-HT injection were significant in both nurse and forager bees, and melatonin similarly exerted a significant suppressive effect, particularly in the nurse bee brain. The lack of significance in some comparisons may reflect the relatively low control levels. Overall, these findings suggest that indolamine signaling remains responsive across developmental stages and may contribute to physiological plasticity associated with worker task allocation.

### 3.3 RNAi-mediated knockdown of Am*5HTR2a* and temporal dynamics

**Figure 3:**
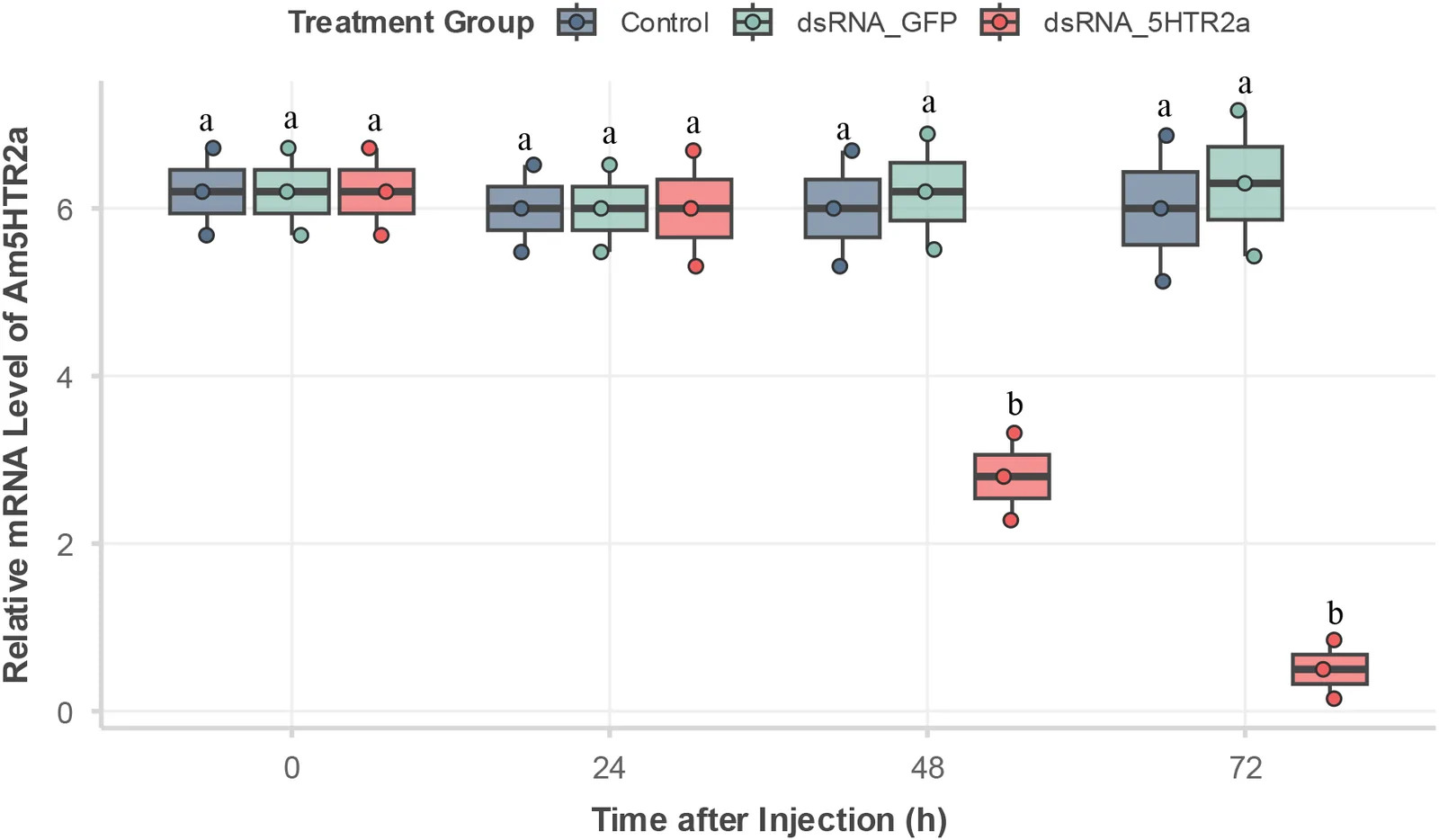
RNAi-mediated knockdown of Am*5HTR2a* expression in the honeybee brain. Relative mRNA levels of Am*5HTR2a* were measured by quantitative PCR (qPCR) in the brains of 11-day-old intact bees (Control) and bees injected with either dsRNA-GFP or dsRNA-Am5*HTR2a*. Total RNA was extracted from brains collected at 0, 24, 48, and 72 h post-injection. Data are presented as mean ± S.E.M. from three independent biological replicates. Statistical analysis was performed using two-way ANOVA followed by Tukey’s Honestly Significant Difference (HSD) *post-hoc* test, with familywise error rate controlled at α = 0.05. Different letters above boxes (a, b) indicate statistically significant differences between treatment groups at each time point.

To functionally validate the role of Am*5HTR2a* in downstream gene regulation, RNA interference (RNAi) was employed to silence receptor expression in the brains of 11-day-old nurse bees. Relative Am*5HTR2a* mRNA levels were monitored at 0, 24, 48, and 72 h post-injection in three treatment groups: intact controls, dsRNA-GFP-injected bees (injection control), and dsRNA-Am*5HTR2a*-injected bees (**Figure 3**; **Supplementary Table 5**).

Two-way ANOVA revealed highly significant main effects of Treatment (*F*_2_,_23_ = 51.391, *p* < 0.001), Time (*F*_3_,_23_ = 18.741, *p* < 0.001), and their interaction (*F*_6_,_23_ = 19.490, *p* < 0.001; **Table 2**). At baseline (0 h) and 24 h post-injection, no significant differences were observed among the three groups, with all groups maintaining expression levels near 6.00–6.20 (**Supplementary Table 5**). However, by 48 h, dsRNA-Am*5HTR2a*-injected bees exhibited a dramatic reduction in expression (2.80 ± 0.52) compared to both control (6.00 ± 0.69) and dsRNA-GFP-injected bees (6.20 ± 0.69; *p* < 0.01; Tukey’s HSD). The knockdown was further intensified at 72 h post-injection, with Am*5HTR2a* expression in dsRNA-treated bees declining to 0.50 ± 0.35, representing a >90% reduction relative to controls (6.00 ± 0.87) and dsRNA-GFP bees (6.30 ± 0.87; *p* < 0.001; **Figure 3**).

**Table 2:** Two-way ANOVA table for relative mRNA levels of Am5HTR2a evaluating main effects of treatment, time, and their interactive effects.

Factor	Df	Sum Sq	Mean Sq	F-value	p-value
Treatment	2	40.145	20.072	51.391	0.000000
Time factor	3	21.960	7.320	18.741	0.000002
Treatment: Time_factor	6	45.675	7.613	19.490	0.000000
Residuals	24	9.374	0.391		

### 3.4 Downstream effect of Am*5HTR2a* knockdown on MRJP1 expression

**Figure 4:**
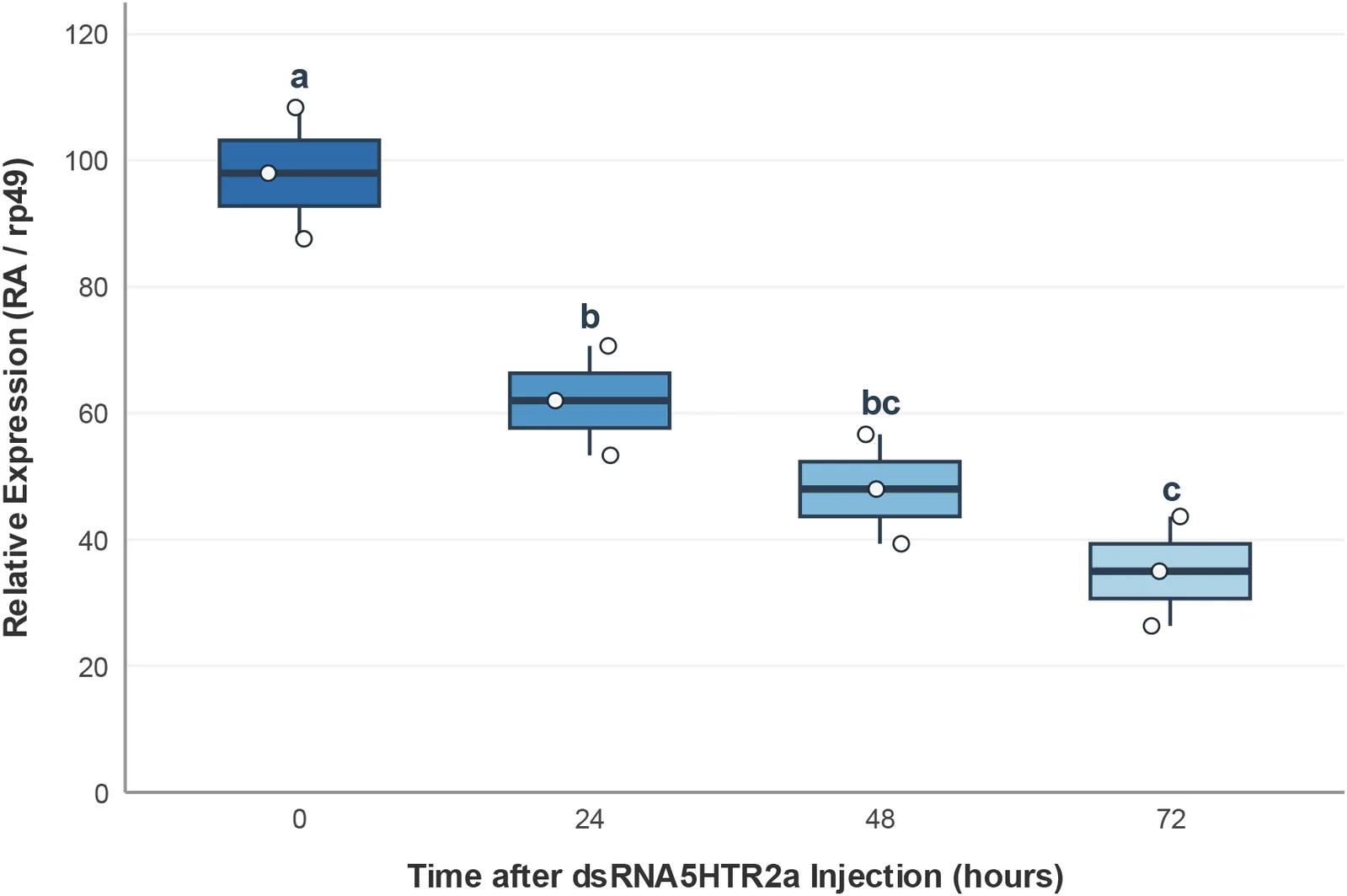
Effect of dsRNA-Am*5HTR2a* (RA) injection on *MRJP1* (*RA*) gene expression in 11-day-old honey bees. Relative expression of the MRJP1 gene was quantified by qPCR amplification of the MRJP1 repetitive region, normalized to the reference gene *rp49*, in bees injected with dsRNA-Am*5HTR2a* and sampled at 0, 24, 48, and 72 h post-injection. Data are presented as median with interquartile range (IQR), with individual replicates shown as open circles. Statistical differences among time points were determined by one-way ANOVA followed by Tukey’s Honestly Significant Difference (HSD) *post-hoc* test, with the familywise error rate controlled at α = 0.05. Different letters above boxes (a, b, bc, c) indicate statistically significant differences between time points.

To assess whether Am*5HTR2a* silencing affected the expression of the downstream effector MRJP1, relative MRJP1 mRNA levels were quantified in dsRNA-Am*5HTR2a*-injected bees over the same post-injection time course (**Figure 4**; **Supplementary Table 6**). One-way ANOVA demonstrated a highly significant effect of time on MRJP1 expression (*F*_3_,_8_ = 26.608, *p* < 0.001; **Table 3**). At 0 h, MRJP1 expression was robust (98.00 ± 10.39), consistent with normal nurse-bee physiology. Expression declined progressively to 62.00 ± 8.66 at 24 h, 48.00 ± 8.66 at 48 h, and 35.00 ± 8.66 at 72 h, representing a cumulative 64.3% reduction from baseline (**Supplementary Table 6**).

**Table 3:** One-way analysis of variance (ANOVA) summary for MRJP1 gene expression.

Source of variation	df	Sum of squares	Mean square	F	p-value
Time (hours)	3	6,644.250	2,214.750	26.608	0.00016
Residuals	8	665.878	83.235		

Tukey’s HSD *post-hoc* comparisons revealed a significant decline between 0 h and 24 h (*p* < 0.05), with further significant reductions observed at 72 h compared to 0 h (*p* < 0.05). The 48-h time point occupied an intermediate position, reflecting the progressive nature of the MRJP1 decline following receptor silencing (**Figure 4**). These findings establish a functional link between Am*5HTR2a* expression and the transcriptional regulation of MRJP1, suggesting that serotonin signaling through this receptor subtype is requisite for sustaining MRJP1 production in nurse bee tissues.

### 3.5 Pharmacological modulation of MRJP1 expression by serotonin and melatonin

**Figure 5:**
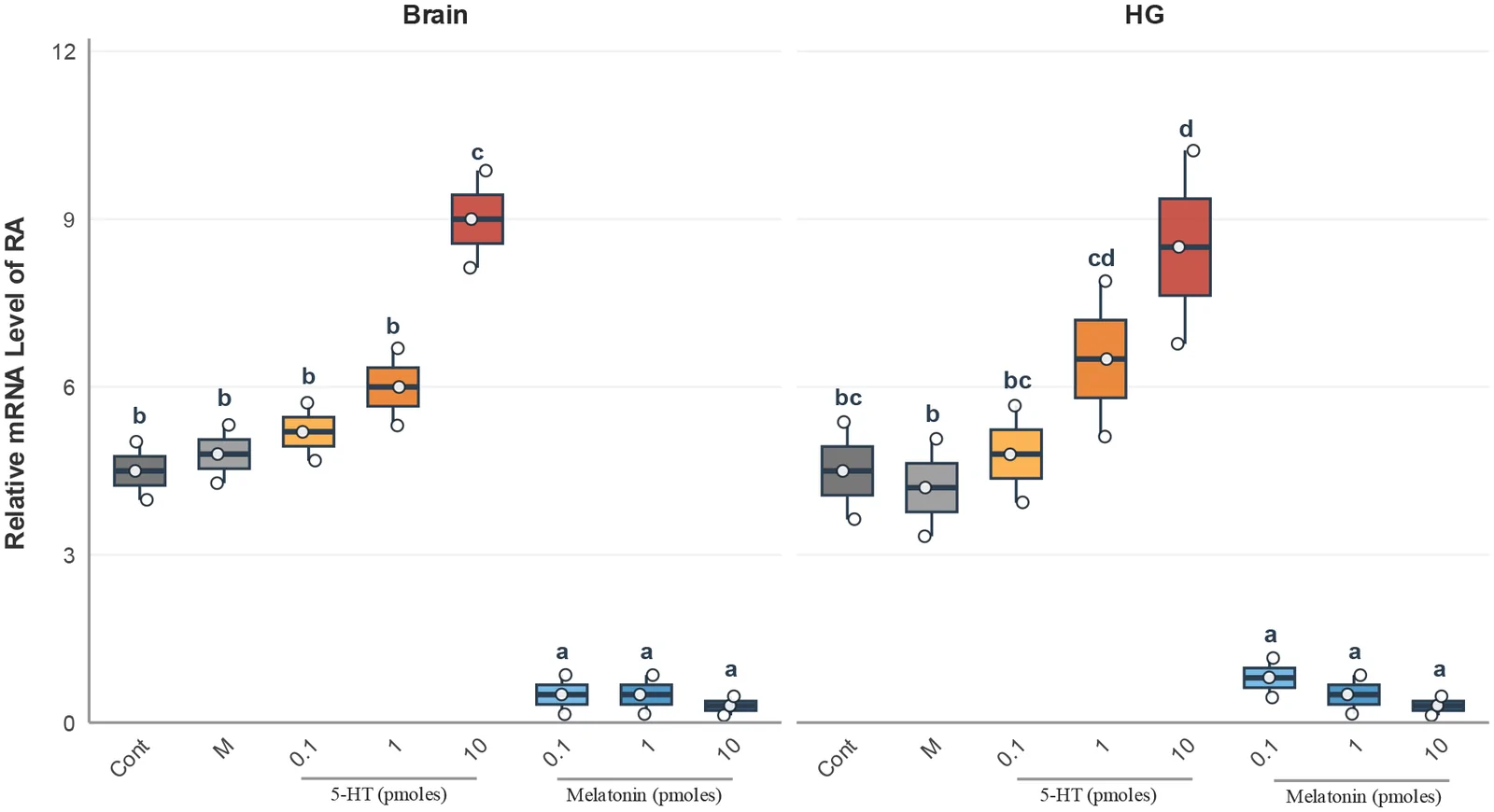
Effects of 5-HT and melatonin injection on *MRJP1* (*RA*) gene expression in the honeybee brain and hypopharyngeal glands. Relative mRNA levels of *MRJP1* were quantified by qPCR in the brain (left panel) and hypopharyngeal glands (HG; right panel) of 11-day-old bees injected with 5-hydroxytryptamine (5-HT) or melatonin at doses of 0.1, 1, or 10 pmoles. Control (Cont.) bees were left untreated; mock-injected (M) bees received 5 µl of distilled water (for 5-HT groups) or 1% ethanol (for melatonin groups). Data are presented as median with interquartile range (IQR), with individual replicates shown as open circles. Statistical analysis was performed using two-way ANOVA followed by Tukey’s Honestly Significant Difference (HSD) *post-hoc* test, with familywise error rate controlled at α = 0.05. Different letters above boxes indicate statistically significant differences between groups within each tissue panel.

To further elucidate the serotonergic regulation of MRJP1, 11-day-old nurse bees were injected with graded doses of 5-HT or melatonin (0.1, 1, and 10 pmoles), and relative MRJP1 mRNA levels were quantified in both the brain and HG (**Figure 5**; **Supplementary Table 7**). Two-way ANOVA revealed significant main effects of Treatment (*F*_7_,_32_ = 92.460, *p* < 0.001) and a non-significant effect of Tissue (*F*_1_,_32_ = 0.150, *p* = 0.701), indicating that pharmacological effects on MRJP1 expression were consistent across tissue types. The Tissue × Treatment interaction was also non-significant (*F*_7_,_32_ = 0.367, *p* = 0.914; **Table 4**).

**Table 4:** Two-way analysis of variance (ANOVA) for the effects of tissue type, pharmacological treatment, and their interaction on relative mRNA expression of MRJP1.

Source	df	SS	MS	F	p-value
Tissue	1	0.092	0.092	0.150	0.70092
Treatment	7	395.923	56.560	92.460	0.00000
Tissue x Treatment	7	1.573	0.225	0.367	0.91447
Residuals	32	19.575	0.612		

In the brain, 5-HT treatment induced a dose-dependent increase in MRJP1 expression. While 0.1 pmol 5-HT produced no significant change relative to controls (5.20 ± 0.52 vs. 4.50 ± 0.52), both the 1 pmol (6.00 ± 0.69) and 10 pmol doses (9.00 ± 0.87) significantly elevated MRJP1 expression (*p* < 0.05; Tukey’s HSD). Conversely, all three doses of melatonin dramatically suppressed MRJP1 expression in the brain to levels of 0.30–0.50, representing a >90% reduction relative to controls (*p* < 0.001; **Supplementary Table 7**). In the HG, an analogous dose-dependent upregulation by 5-HT was observed, with significant increases at 1 pmol (6.50 ± 1.39) and 10 pmol (8.50 ± 1.73) relative to controls (4.50 ± 0.87; *p* < 0.05). Melatonin similarly suppressed HG MRJP1 expression to 0.30–0.80 across all doses (**Figure 5**).

### 3.6 Age-dependent dose–response of MRJP1 to 5-HT treatment

**Figure 6:**
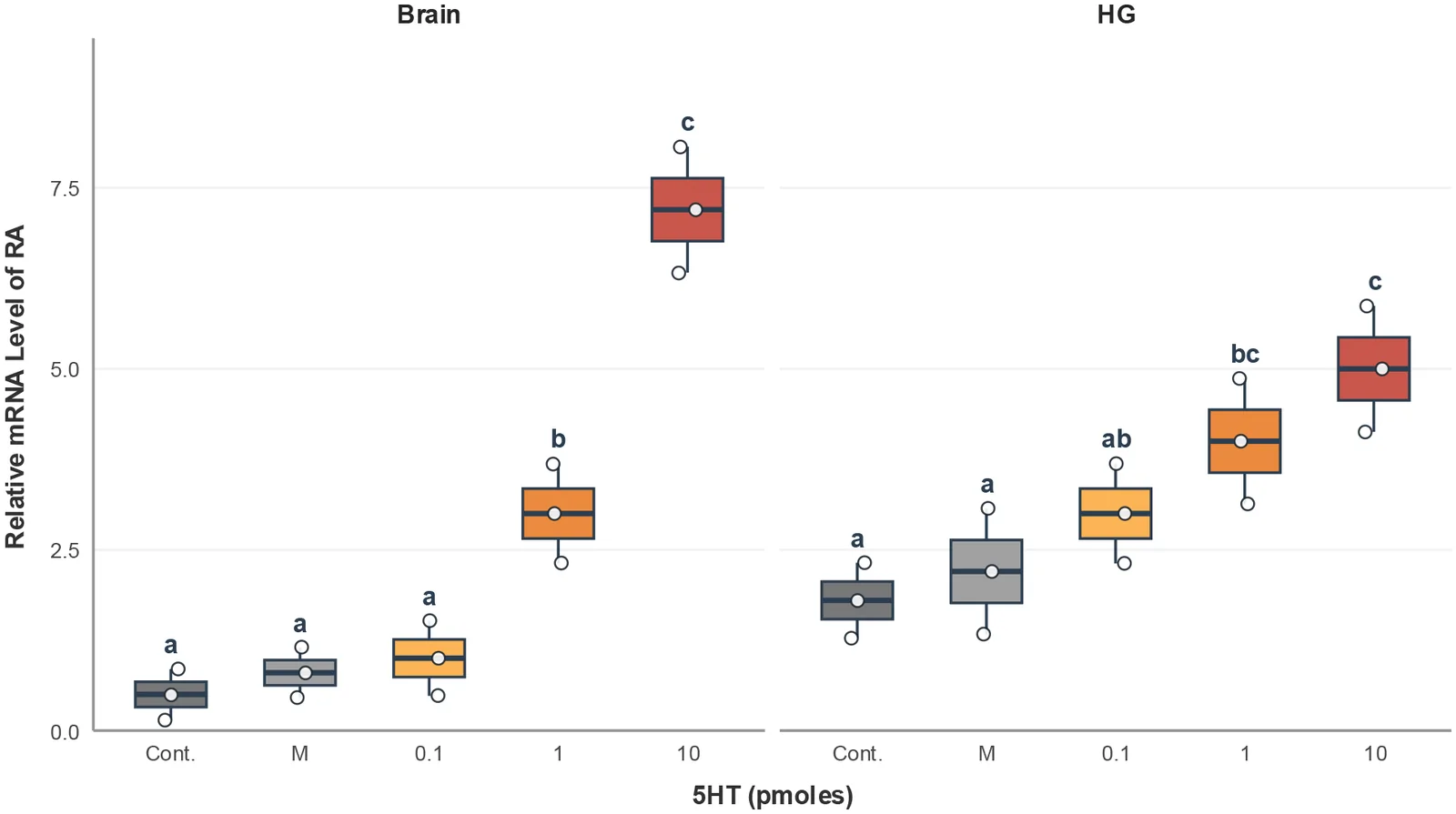
Relative *MRJP1* (*RA*) mRNA expression levels in the brain and hypopharyngeal gland (HG) of 21-day-old honey bees following 5-HT treatment. Bees were subjected to three doses of 5-HT (0.1, 1, and 10 pmol). Cont., untreated control; M, mock injection with 5 µL of distilled water (vehicle control); 0.1, 1, and 10, groups injected with 5-HT dissolved in the vehicle. Boxplots display the median (center line), 25th–75th percentiles (bounds of box), and whiskers (1.5 × interquartile range), with individual biological replicates overlaid as jittered points (n=3 per group). Different letters above the boxes denote statistically significant differences within each tissue type as determined by Two-Way ANOVA followed by Tukey’s *post-hoc* test (p<0.05).

To determine whether the 5-HT-mediated upregulation of MRJP1 is specific to nurse bees or persists into the forager stage, 21-day-old forager bees were injected with 5-HT at 0.1, 1, and 10 pmoles, and relative MRJP1 levels were measured in the brain and HG (**Figure 6**; **Supplementary Table 8**). Two-way ANOVA revealed significant main effects of Tissue (*F*_1_,_20_ = 7.712, *p* = 0.012) and Treatment (*F*_3_,_20_ = 51.683, *p* < 0.001), as well as a significant Tissue × Treatment interaction (*F*_3_,_20_ = 8.687, *p* < 0.001; **Table 5**), indicating tissue-specific dose–response dynamics.

**Table 5:** Two-way analysis of variance (ANOVA) of relative MRJP1 mRNA expression levels in honey bee brain and hypopharyngeal gland (HG) tissues following 5-HT treatment.

Source	df	SS	MS	F	p-value
Tissue	1	3.675	3.675	7.712	0.01163
Treatment	4	98.520	24.630	51.683	0.00000
Tissue x Treatment	4	16.560	4.140	8.687	0.00031
Residuals	20	9.531	0.477		

In the forager brain, baseline MRJP1 expression was markedly lower than in nurse bees (0.50 ± 0.35 vs. 4.50 ± 0.52 at Day 11), consistent with the age-dependent decline in MRJP1 transcription. Notably, 5-HT was able to rescue MRJP1 expression in a dose-dependent manner: the 0.1 pmol dose produced a modest, non-significant increase (1.00 ± 0.52), whereas 1 pmol (3.00 ± 0.69) and 10 pmol (7.20 ± 0.87) significantly upregulated MRJP1 expression (*p* < 0.05; Tukey’s HSD). In the forager HG, a similar dose-dependent pattern was observed, with significant increases at 1 pmol (4.00 ± 0.87) and 10 pmol (5.00 ± 0.87) compared to controls (1.80 ± 0.52; *p* < 0.05; **Figure 6**; **Supplementary Table 8**).

### 3.7 Effect of serotonergic depletion on MRJP1 expression

**Figure 7:**
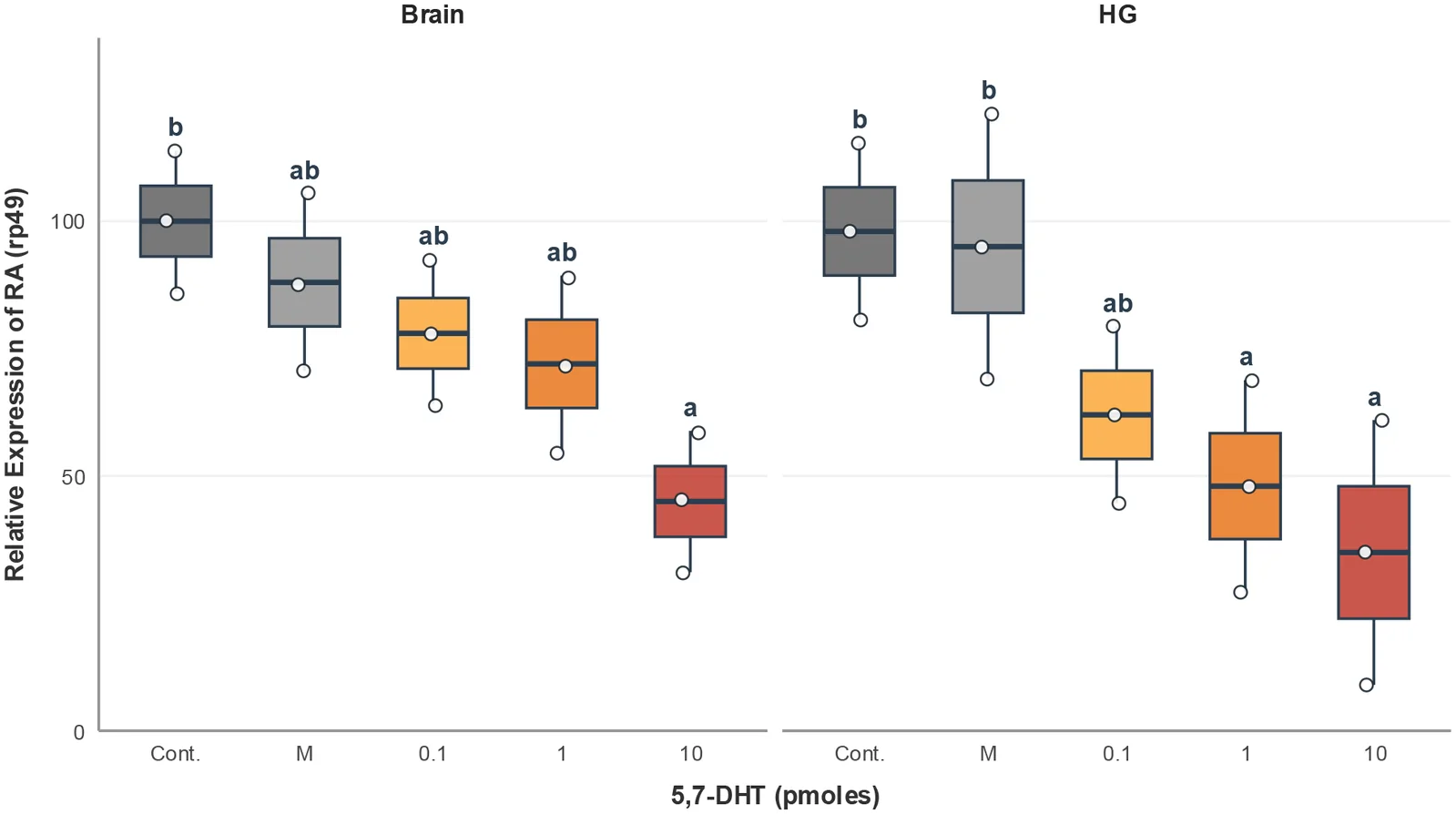
Relative expression level of *MRJP1* (*RA*) (normalized to *rp49*) in 11-day-old honey bees after depletion of indolamines by 5,7-DHT injection. Eleven-day-old bees were injected with 5,7-DHT at three doses (0.1, 1, and 10 pmoles). Cont., untreated. M, mock injection with 5 μl of distilled water. 5,7-DHT, injected with 5,7-DHT dissolved in the same volume as in the mock. Data are presented for the brain (left panel) and hypopharyngeal gland (HG; right panel). Box boundaries define the interquartile range (IQR), the horizontal line represents the median, and whiskers extend to 1.5×IQR. White circles represent individual biological replicates (n=3 per group). Different letters above the boxes indicate statistically significant differences between treatments within each tissue based on a Two-Way ANOVA followed by a Tukey-adjusted *post-hoc* comparison (p<0.05).

To evaluate the consequences of endogenous serotonin depletion on MRJP1 expression, 11-day-old nurse bees were injected with the selective serotonergic neurotoxin 5,7-dihydroxytryptamine (5,7-DHT) at 0.1, 1, and 10 pmoles. Relative MRJP1 mRNA levels were quantified in both the brain and HG (**Figure 7**; **Supplementary Table 9**). Two-way ANOVA revealed a significant main effect of Treatment (*F*_3_,_20_ = 9.617, *p* < 0.001), while the main effect of Tissue (*F*_1_,_20_ = 1.707, *p* = 0.206) and the Tissue × Treatment interaction (*F*_3_,_20_ = 0.611, *p* = 0.659) were non-significant (**Table 6**), indicating that 5,7-DHT affected MRJP1 expression uniformly across tissues.

**Table 6:** Two-way analysis of variance (ANOVA) of relative MRJP1 expression levels (normalized to rp49) in honey bee brain and hypopharyngeal gland (HG) tissues following 5,7-DHT treatment.

Source	df	SS	MS	F	p-value
Tissue	1	607.500	607.500	1.707	0.20614
Treatment	4	13,687.200	3,421.800	9.617	0.00017
Tissue x Treatment	4	870.000	217.500	0.611	0.65930
Residuals	20	7,115.915	355.796		

In the brain, MRJP1 expression declined from 100.00 ± 13.86 in controls to 78.00 ± 13.86, 72.00 ± 17.32, and 45.00 ± 13.86 at 0.1, 1, and 10 pmol 5,7-DHT, respectively, with the highest dose producing a significant 55% reduction (*p* < 0.05; Tukey’s HSD). In the HG, a more pronounced dose-dependent decline was observed: from 98.00 ± 17.32 in controls to 62.00 ± 17.32 (0.1 pmol), 48.00 ± 20.78 (1 pmol), and 35.00 ± 25.98 (10 pmol). The highest dose of 5,7-DHT elicited a significant 64.3% reduction in MRJP1 expression relative to controls (*p* < 0.05; **Figure 7**; **Supplementary Table 9**). These findings provide converging pharmacological evidence that endogenous serotonin contributes to the maintenance of MRJP1 transcription in the honeybee, and that disruption of serotonergic signaling, whether by receptor silencing or neurotransmitter depletion, leads to a profound suppression of MRJP1 gene expression.

## 4 Discussion

The present study identifies Am*5HTR2a* as a serotonin receptor subtype whose expression closely tracks adult behavioral maturation and MRJP1 abundance in *Apis mellifera* workers. Among the receptor subtypes examined, Am*5HTR2a* showed the clearest age-dependent pattern, increasing during early adult life, peaking during the nurse bee phase associated with MRJP1 production, and declining as workers transitioned to foraging. This temporal profile paralleled the period of active HG secretory activity and royal jelly production, supporting a link between serotonergic signaling and the physiological state underlying brood-care behavior. Pharmacological and integrative molecular evidence indicates that serotonergic pathways modulate feeding-related neural circuits and peripheral digestive physiology, while transcriptomic studies of nutrition-sensitive tissues, including HG, support a conserved role of neuromodulatory–endocrine systems in nutrient processing and provisioning (Alaux et al., 2011; French et al., 2014; Peng et al., 2024; D’Emilio et al., 2025; Maigoro et al., 2025). In honey bees, nutritional state and endocrine signaling pathways, particularly vitellogenin and its receptor-mediated uptake in HG, are tightly coupled to gland development and brood-food production, reinforcing the integration between metabolism, reproduction-related pathways, and social behavior (Harwood et al., 2019; Dohanik et al., 2024; Güneşdoğdu et al., 2024; D’Emilio et al., 2025). Age-related behavioral specialization is further accompanied by coordinated endocrine, metabolic, and neural remodeling during the nurse-to-forager transition (Hamilton et al., 2019; Schilcher and Scheiner, 2023; Barbero and Casacci, 2025). The restricted developmental dynamics observed for Am*5HTR2a*, compared with the relatively stable expression of the other receptor subtypes examined here, suggest that this receptor may be particularly involved in processes associated with nursing physiology while remaining part of the broader serotonergic signaling network.

Manipulation of serotonergic tone produced corresponding shifts in both Am5HTR2a and MRJP1 transcript abundance. Serotonin treatment increased receptor expression and elevated MRJP1 levels in both the brain and HG, whereas melatonin treatment produced the opposite trend. Similarly, reducing serotonergic signaling through either RNAi-mediated knockdown of Am5HTR2a or depletion of endogenous serotonin using 5,7-DHT consistently lowered MRJP1 expression. Although these experiments do not demonstrate direct transcriptional regulation, the concordance between pharmacological manipulations and RNAi supports a functional association between serotonergic signaling and MRJP1-related physiology. Notably, serotonin retained the capacity to elevate MRJP1 expression even in forager bees, despite their substantially lower baseline levels. This observation suggests that components of the serotonergic response system remain active beyond the peak nursing period and may not be irreversibly silenced during behavioral maturation. This residual responsiveness implies that the molecular machinery linking serotonin to MRJP1 expression is retained in forager tissues, possibly enabling context-dependent reactivation of nurse-like physiology in response to colony demands (Schilcher and Scheiner, 2023). The dose-dependent nature of this effect further suggests that threshold levels of serotonergic tone, acting in concert with melatonergic tone, may function as a switch influencing MRJP1 output across worker states. The succession of these distinct, stage-specific states, together with their characteristic behavioral repertoires, constitutes honey bee temporal polyethism (Johnson, 2008; Schilcher and Scheiner, 2023). These findings are consistent with the established role of serotonin as an important regulator of worker physiological state in honey bees, where serotonergic signaling influences behavioral specialization and food-related activities through receptor-mediated mechanisms (French et al., 2014; Carvalho da Silva et al., 2024). Melatonin appears to antagonize this pathway and may help sharpen the division of labor (Yang et al., 2007; Nahar et al., 2026). As demonstrated by Izawa et al. (2023) and Nahar et al. (2026), AANAT regulates the balance of these indoleamines. Consistent with its designation as a circadian timing enzyme (“Timezyme”), AANAT provides a mechanistic link between temporal regulation and indoleamine metabolism (Klein, 2007). AmAANAT transcription is under circadian control, and RNAi-mediated knockdown of the enzyme altered the behavioral transition (Nahar et al., 2026). Thus, the social structure of this species retains flexibility, allowing the transition to remain reversible when colony conditions require it (Schilcher and Scheiner, 2023; Nahar et al., 2026). In this way, behavior–metabolism integration is maintained (Hamilton et al., 2019; Schilcher and Scheiner, 2023). Notably, serotonin-treated foragers exhibited elevated MRJP1 expression despite retaining their forager status, indicating that molecular responsiveness can persist without an immediate behavioral transition.

The pronounced expression of Am5HTR2a within the HG is particularly notable given the gland’s central role in brood food secretion and worker task specialization. HG development and activity are tightly linked to worker age and colony nutritional demands, with nurse bees displaying enlarged secretory acini and elevated royal jelly protein synthesis relative to foragers (Knecht and Kaatz, 1990; Ueno et al., 2009). Biogenic amines, including serotonin, dopamine, and octopamine, have long been implicated in regulating behavioral maturation and sensory responsiveness in honey bees and other eusocial insects (Sasaki and Watanabe, 2022; Carvalho da Silva et al., 2024). Previous characterization of honey bee serotonin receptors demonstrated especially strong expression of 5-HT_2_ receptors in exocrine tissues, including the HG, suggesting a potential role in glandular regulation (Thamm et al., 2013). The present findings are consistent with that interpretation and further suggest that serotonergic responsiveness in peripheral tissues may contribute to molecular processes associated with HG activity during brood care. More broadly, these observations align with increasing evidence that division of labor in honey bees emerges through interactions among neuromodulators, endocrine pathways, nutritional state, and social context rather than through the action of a single regulatory factor (Schilcher and Scheiner, 2023). Notably, the 5-HT_2_ receptor subtype identified here is a Gαq/11-coupled receptor capable of mobilizing intracellular Ca²^+^ stores (Thamm et al., 2013), a signaling cascade known to stimulate secretory activity in insect exocrine glands. This molecular architecture positions Am5HTR2a as a plausible direct mediator of serotonin-induced MRJP1 synthesis, potentially via Ca²^+^-dependent transcription factors that remain to be identified. Although the present data do not identify the downstream effectors linking *Am5HTR2a* signaling to *MRJP1* expression, Ca²^+^-responsive transcriptional regulators such as CREB are plausible candidates based on their established roles in Ca²^+^-dependent gene regulation (Mayr and Montminy, 2001; Mellström et al., 2008). Future promoter-reporter or transcription-factor-binding assays will be required to confirm whether these factors mediate Am5HTR2a-dependent MRJP1 regulation.

The relationship between serotonergic signaling and temporal polyethism likely occurs within a broader endocrine framework involving JH, vitellogenin, and metabolic state. JH has traditionally been viewed as a major regulator of behavioral maturation in honey bees, although more recent work suggests that its role is strongly integrated with nutritional physiology and vitellogenin-mediated feedback networks (Wegener et al., 2013; Schilcher and Scheiner, 2023). Vitellogenin itself is closely associated with nursing behavior and brood food production and is thought to interact with multiple neuroendocrine systems that coordinate worker task allocation (Unnikrishnan et al., 2026). While the present study did not directly examine these pathways, the age-dependent expression of Am5HTR2a and its responsiveness to serotonergic manipulation raise the possibility that serotonin signaling may intersect with broader endocrine circuits involved in maintaining the nurse phenotype. Intersection between serotonin and JH/vitellogenin networks may occur at the level of the HG, where local serotonergic tone could modulate the sensitivity of secretory cells to systemic endocrine signals. Future studies employing dual manipulations of serotonin and JH pathways will be needed to test whether these systems act synergistically or independently to stabilize the nurse state.

A few methodological considerations are relevant to the interpretation of these findings. RNAi-mediated knockdown of *Am5HTR2a* reduced both receptor and *MRJP1* transcripts; however, *Am5HTR2b* and the other receptor subtypes were not re-quantified following dsRNA treatment, so potential off-target effects cannot be formally excluded. The distinct ontogenetic expression profiles support the specificity of the qPCR assays, while complementary receptor-specific approaches would provide further validation of the role of *Am5HTR2a* signaling in *MRJP1* regulation. The relatively small number of biological replicates (n = 3 per group) should be considered when interpreting the findings. In addition, *rp49* was not formally validated across the developmental HG series, and behavioral state was inferred from chronological age. The indoleamine doses were selected as a graded pharmacological series rather than calibrated to endogenous concentrations. Finally, we acknowledge that our conclusions regarding Am5HTR2a receptor dynamics are based solely on mRNA abundance measured by RT-qPCR, without protein-level validation. Future proteomic analyses are therefore warranted to determine whether these transcriptional changes translate into corresponding changes in receptor protein abundance (Hora et al., 2018; Arad et al., 2025).

Overall, the data indicate that Am5HTR2a expression is dynamically associated with worker maturation and with MRJP1 abundance in both neural and exocrine tissues of the honey bee. The findings support an association between serotonergic signaling and physiological features characteristic of the nurse bee state and suggest that Am5HTR2a may represent one component through which neuromodulatory signaling interfaces with HG function during temporal polyethism. Future studies combining receptor localization, protein-level analyses, and functional behavioral assays should help clarify how serotonergic pathways contribute to the coordination of glandular physiology and worker behavioral specialization. Extending this work to other eusocial species with different degrees of reproductive division of labor would help clarify whether serotonin-mediated regulation of brood-food glands is a general feature of social insect evolution or a derived trait of the *Apis* lineage.

## 5 Conclusion

Our findings identify Am5HTR2a as a serotonin receptor subtype whose expression is closely coupled to the nurse-bee stage and to MRJP1 transcription in both brain and hypopharyngeal gland tissue. Convergent evidence from developmental profiling, pharmacological manipulation, endogenous neurotransmitter depletion, and RNAi supports a serotonergic signaling axis that sustains the molecular and physiological state associated with brood-food production. The persistence of serotonergic responsiveness in foragers, together with its dose-dependent capacity to restore MRJP1 expression in that stage, indicates that this pathway is not irreversibly silenced during behavioral maturation but may remain poised for context-dependent reactivation in response to colony demand. Melatonin, produced downstream of serotonin through the arylalkylamine pathway, consistently opposed these effects, indicating a reciprocal relationship between these two indoleamine signals in shaping the nurse-to-forager transition. Together, these observations place neuromodulatory signaling within the broader framework of temporal polyethism, alongside established endocrine regulators such as juvenile hormone and vitellogenin. Future work combining receptor localization, protein-level analyses, and *in vivo* behavioral assays will be required to define the downstream transcriptional targets of Am5HTR2a in secretory tissues and to determine whether serotonin-mediated regulation of brood-food glands is a conserved feature of social insect biology or a derived specialization of the *Apis* lineage.

## Data Availability

The original contributions presented in the study are included in the article/**Supplementary Material**. Further inquiries can be directed to the corresponding author.
