# Supplementary material for: The serotonin receptor Am5HTR2a links neuromodulation to MRJP1 expression and temporal polyethism in honey bees
Source: Front Physiol. 2026 Sep 9;17:1926478. doi: 10.3389/fphys.2026.1926478 (PMC13597315; doi:10.3389/fphys.2026.1926478)
Supplement: Supplementary file 1 [file Table1.docx]

**Table S1.** Primers used in this study

| **Name** | **Sequence (5’ → 3’)** |
| --- | --- |
| Am5HTR2a-T7-F | **TAATACGACTCACTATAGGGAGA**CTACGGAGGATCTGACTATCTCTCTTG |
| Am5HTR2a-T7-R | **TAATACGACTCACTATAGGGAGA**AGAGACAAAAGGAAGTAATTGGTGA |
| Am5HTR1a-F | CTACCCCTGTTGGTTATTCTATTCC |
| Am5HTR1a-R | GTTGTAAGACGACGATTTCTCAGG |
| Am5HTR2a-F | TCTTCATGTTCGTGCTCTGC |
| Am5HTR2a-R | CCTGGAACACTTGCACTTCA |
| Am5HTR2b-F | ACGAGCTGAATTCCGTTGTC |
| Am5HTR2b-R | GCTGCGTACTGTCGTTTGGT |
| Am5HTR7-F | GTGATGTGATAGTTCATTGCGTTAC |
| Am5HTR7-R | AGTCATTATCCGGATTGAACGTGT |
| GFP-T7-F | **TAATACGACTCACTATAGGGAGA**CCTGAAGTTCATCTGCACCAC |
| GFP-T7-R | **TAATACGACTCACTATAGGGAGA**AACGAACTCCAGCAGGACCAT |
| MRJP1-F | AATGTAAACGAATTGATATTGAACA |
| MRJP1-R | ATTCATAATGGAAAGAAATTTCGAG |
| RP49-F | TCGTCACCAGAGTGATCGTT |
| RP49-R | CCCATGAGCAATTTCAGCAC |

*Underlined sequences represent the T7 promoter. Receptor nomenclature and primer design were based on NCBI RefSeq records (Am5HTR2a: NM_001202460.1; accessed 15 May 2026).*

**Table S2.** Descriptive statistics of relative mRNA levels of Am5HTRs in the brain

| **Gene** | **Day** | **N** | **Mean** | **SEM** | **SD** | **Median** | **Min** | **Max** |
| --- | --- | --- | --- | --- | --- | --- | --- | --- |
| 5HTR1 | 3 | 3 | 0.500 | 0.098 | 0.170 | 0.500 | 0.330 | 0.670 |
| 5HTR1 | 6 | 3 | 0.500 | 0.098 | 0.170 | 0.500 | 0.330 | 0.670 |
| 5HTR1 | 11 | 3 | 0.800 | 0.098 | 0.170 | 0.800 | 0.630 | 0.970 |
| 5HTR1 | 16 | 3 | 0.700 | 0.098 | 0.170 | 0.700 | 0.530 | 0.870 |
| 5HTR1 | 21 | 3 | 0.500 | 0.098 | 0.170 | 0.500 | 0.330 | 0.670 |
| 5HTR1 | 23 | 3 | 0.400 | 0.098 | 0.170 | 0.400 | 0.230 | 0.570 |
| 5HTR7 | 3 | 3 | 0.700 | 0.202 | 0.350 | 0.700 | 0.350 | 1.050 |
| 5HTR7 | 6 | 3 | 0.800 | 0.202 | 0.350 | 0.800 | 0.450 | 1.150 |
| 5HTR7 | 11 | 3 | 1.200 | 0.202 | 0.350 | 1.200 | 0.850 | 1.550 |
| 5HTR7 | 16 | 3 | 1.500 | 0.300 | 0.520 | 1.500 | 0.980 | 2.020 |
| 5HTR7 | 21 | 3 | 1.000 | 0.202 | 0.350 | 1.000 | 0.650 | 1.350 |
| 5HTR7 | 23 | 3 | 0.700 | 0.202 | 0.350 | 0.700 | 0.350 | 1.050 |
| 5HTR2a | 3 | 3 | 0.600 | 0.098 | 0.170 | 0.600 | 0.430 | 0.770 |
| 5HTR2a | 6 | 3 | 2.500 | 0.300 | 0.520 | 2.500 | 1.980 | 3.020 |
| 5HTR2a | 11 | 3 | 6.300 | 0.502 | 0.870 | 6.300 | 5.430 | 7.170 |
| 5HTR2a | 16 | 3 | 5.800 | 0.398 | 0.690 | 5.800 | 5.110 | 6.490 |
| 5HTR2a | 21 | 3 | 1.000 | 0.202 | 0.350 | 1.000 | 0.650 | 1.350 |
| 5HTR2a | 23 | 3 | 0.500 | 0.150 | 0.260 | 0.500 | 0.240 | 0.760 |
| 5HTR2b | 3 | 3 | 2.800 | 0.300 | 0.520 | 2.800 | 2.280 | 3.320 |
| 5HTR2b | 6 | 3 | 2.500 | 0.202 | 0.350 | 2.500 | 2.150 | 2.850 |
| 5HTR2b | 11 | 3 | 2.500 | 0.300 | 0.520 | 2.500 | 1.980 | 3.020 |
| 5HTR2b | 16 | 3 | 2.300 | 0.300 | 0.520 | 2.300 | 1.780 | 2.820 |
| 5HTR2b | 21 | 3 | 2.200 | 0.300 | 0.520 | 2.200 | 1.680 | 2.720 |
| 5HTR2b | 23 | 3 | 2.000 | 0.300 | 0.520 | 2.000 | 1.480 | 2.520 |

**Table S3.** Descriptive statistics of relative expression (5HTR2a/rp49) in HG

| **Day** | **N** | **Mean** | **SEM** | **SD** | **Median** | **Min** | **Max** |
| --- | --- | --- | --- | --- | --- | --- | --- |
| 3 | 3 | 0.00 | 0.00 | 0.00 | 0.00 | 0.00 | 0.00 |
| 6 | 3 | 58.00 | 5.00 | 8.66 | 58.00 | 49.34 | 66.66 |
| 11 | 3 | 89.00 | 7.00 | 12.12 | 89.00 | 76.88 | 101.12 |
| 16 | 3 | 98.00 | 5.00 | 8.66 | 98.00 | 89.34 | 106.66 |
| 21 | 3 | 0.00 | 0.00 | 0.00 | 0.00 | 0.00 | 0.00 |
| 23 | 3 | 0.00 | 0.00 | 0.00 | 0.00 | 0.00 | 0.00 |

**Table S4.** Descriptive statistics of Am5HTR2a relative mRNA levels in *Apis* *mellifera* brain and hypopharyngeal gland (HG). Values represent mean +/- SD. SE = standard error; N = number of biological replicates

| **Tissue** | **Age (days)** | **Treatment** | **N** | **Mean +/- SD** | **SE** | **Min** | **Max** |
| --- | --- | --- | --- | --- | --- | --- | --- |
| Brain | Day 11 (Nurse) | Control | 3 | 6.3 +/- 0.69 | 0.398 | 5.61 | 6.99 |
| Brain | Day 11 (Nurse) | 5HT | 3 | 7.8 +/- 0.87 | 0.502 | 6.93 | 8.67 |
| Brain | Day 11 (Nurse) | Melatonin | 3 | 1.2 +/- 0.35 | 0.202 | 0.85 | 1.55 |
| Brain | Day 21 (Forager) | Control | 3 | 4.2 +/- 0.69 | 0.398 | 3.51 | 4.89 |
| Brain | Day 21 (Forager) | 5HT | 3 | 8 +/- 0.87 | 0.502 | 7.13 | 8.87 |
| HG | Day 11 (Nurse) | Control | 3 | 3.5 +/- 0.87 | 0.502 | 2.63 | 4.37 |
| HG | Day 11 (Nurse) | 5HT | 3 | 6.5 +/- 0.87 | 0.502 | 5.63 | 7.37 |
| HG | Day 11 (Nurse) | Melatonin | 3 | 2.5 +/- 0.52 | 0.300 | 1.98 | 3.02 |
| HG | Day 21 (Forager) | Control | 3 | 3 +/- 0.52 | 0.300 | 2.48 | 3.52 |
| HG | Day 21 (Forager) | 5HT | 3 | 4 +/- 0.69 | 0.398 | 3.31 | 4.69 |

**Table S5.** Descriptive Statistical Analysis by Treatment and Time Summary of Relative mRNA levels of Am5HTR2a, presented as Mean, Standard Deviation (SD), Standard Error of the Mean (SEM), Median, Minimum, Maximum, and 95% Confidence Intervals

| **Treatment Group** | **Time (h)** | **N** | **Mean** | **SD** | **SEM** | **Median** | **Min** | **Max** | **95% CI Lower** | **95% CI Upper** |
| --- | --- | --- | --- | --- | --- | --- | --- | --- | --- | --- |
| Control | 0 | 3 | 6.200 | 0.520 | 0.300 | 6.200 | 5.680 | 6.720 | 4.908 | 7.492 |
| Control | 24 | 3 | 6.000 | 0.520 | 0.300 | 6.000 | 5.480 | 6.520 | 4.708 | 7.292 |
| Control | 48 | 3 | 6.000 | 0.690 | 0.398 | 6.000 | 5.310 | 6.690 | 4.286 | 7.714 |
| Control | 72 | 3 | 6.000 | 0.870 | 0.502 | 6.000 | 5.130 | 6.870 | 3.839 | 8.161 |
| dsRNA_GFP | 0 | 3 | 6.200 | 0.520 | 0.300 | 6.200 | 5.680 | 6.720 | 4.908 | 7.492 |
| dsRNA_GFP | 24 | 3 | 6.000 | 0.520 | 0.300 | 6.000 | 5.480 | 6.520 | 4.708 | 7.292 |
| dsRNA_GFP | 48 | 3 | 6.200 | 0.690 | 0.398 | 6.200 | 5.510 | 6.890 | 4.486 | 7.914 |
| dsRNA_GFP | 72 | 3 | 6.300 | 0.870 | 0.502 | 6.300 | 5.430 | 7.170 | 4.139 | 8.461 |
| dsRNA_5HTR2a | 0 | 3 | 6.200 | 0.520 | 0.300 | 6.200 | 5.680 | 6.720 | 4.908 | 7.492 |
| dsRNA_5HTR2a | 24 | 3 | 6.000 | 0.690 | 0.398 | 6.000 | 5.310 | 6.690 | 4.286 | 7.714 |
| dsRNA_5HTR2a | 48 | 3 | 2.800 | 0.520 | 0.300 | 2.800 | 2.280 | 3.320 | 1.508 | 4.092 |
| dsRNA_5HTR2a | 72 | 3 | 0.500 | 0.350 | 0.202 | 0.500 | 0.150 | 0.850 | -0.369 | 1.369 |

**Table S6.** Descriptive Statistics of MRJP1 Gene Expression (Relative_Expression_ MRJP1_rp49) across Time points

| **Time (hours)** | **N** | **Mean** | **SD** | **Standard Error** | **Median** | **Minimum** | **Maximum** | **95% CI Lower** | **95% CI Upper** | **Time_Factor** | **Tukey Group** |
| --- | --- | --- | --- | --- | --- | --- | --- | --- | --- | --- | --- |
| 0 | 3 | 98.000 | 10.390 | 5.999 | 98.000 | 87.610 | 108.390 | 72.190 | 123.810 | 0 | a |
| 24 | 3 | 62.000 | 8.660 | 5.000 | 62.000 | 53.340 | 70.660 | 40.487 | 83.513 | 24 | b |
| 48 | 3 | 48.000 | 8.660 | 5.000 | 48.000 | 39.340 | 56.660 | 26.487 | 69.513 | 48 | bc |
| 72 | 3 | 35.000 | 8.660 | 5.000 | 35.000 | 26.340 | 43.660 | 13.487 | 56.513 | 72 | c |

**Table S7.** Descriptive Statistics of Relative mRNA Level of MRJP1 by Tissue and Treatment

| **Tissue** | **Treatment** | **N** | **Mean** | **SD** | **SE** | **Median** | **Min** | **Max** | **95% CI Lower** | **95% CI Upper** | **Tukey Group** |
| --- | --- | --- | --- | --- | --- | --- | --- | --- | --- | --- | --- |
| Brain | Control | 3 | 4.500 | 0.520 | 0.300 | 4.500 | 3.980 | 5.020 | 3.208 | 5.792 | b |
| Brain | Vehicle | 3 | 4.800 | 0.520 | 0.300 | 4.800 | 4.280 | 5.320 | 3.508 | 6.092 | b |
| Brain | 5HT_0.1 | 3 | 5.200 | 0.520 | 0.300 | 5.200 | 4.680 | 5.720 | 3.908 | 6.492 | b |
| Brain | 5HT_1 | 3 | 6.000 | 0.690 | 0.398 | 6.000 | 5.310 | 6.690 | 4.286 | 7.714 | b |
| Brain | 5HT_10 | 3 | 9.000 | 0.870 | 0.502 | 9.000 | 8.130 | 9.870 | 6.839 | 11.161 | c |
| Brain | Melatonin_0.1 | 3 | 0.500 | 0.350 | 0.202 | 0.500 | 0.150 | 0.850 | -0.369 | 1.369 | a |
| Brain | Melatonin_1 | 3 | 0.500 | 0.350 | 0.202 | 0.500 | 0.150 | 0.850 | -0.369 | 1.369 | a |
| Brain | Melatonin_10 | 3 | 0.300 | 0.170 | 0.098 | 0.300 | 0.130 | 0.470 | -0.122 | 0.722 | a |
| HG | Control | 3 | 4.500 | 0.870 | 0.502 | 4.500 | 3.630 | 5.370 | 2.339 | 6.661 | bc |
| HG | Vehicle | 3 | 4.200 | 0.870 | 0.502 | 4.200 | 3.330 | 5.070 | 2.039 | 6.361 | b |
| HG | 5HT_0.1 | 3 | 4.800 | 0.870 | 0.502 | 4.800 | 3.930 | 5.670 | 2.639 | 6.961 | bc |
| HG | 5HT_1 | 3 | 6.500 | 1.390 | 0.803 | 6.500 | 5.110 | 7.890 | 3.047 | 9.953 | cd |
| HG | 5HT_10 | 3 | 8.500 | 1.730 | 0.999 | 8.500 | 6.770 | 10.230 | 4.202 | 12.798 | d |
| HG | Melatonin_0.1 | 3 | 0.800 | 0.350 | 0.202 | 0.800 | 0.450 | 1.150 | -0.069 | 1.669 | a |
| HG | Melatonin_1 | 3 | 0.500 | 0.350 | 0.202 | 0.500 | 0.150 | 0.850 | -0.369 | 1.369 | a |
| HG | Melatonin_10 | 3 | 0.300 | 0.170 | 0.098 | 0.300 | 0.130 | 0.470 | -0.122 | 0.722 | a |

**Table S8.** Descriptive Statistics of Relative mRNA Level of MRJP1 by Tissue and Treatment

| **Tissue** | **Treatment** | **N** | **Mean** | **SD** | **SE** | **Median** | **Min** | **Max** | **95% CI Lower** | **95% CI Upper** | **Tukey Group** |
| --- | --- | --- | --- | --- | --- | --- | --- | --- | --- | --- | --- |
| Brain | Control | 3 | 0.500 | 0.350 | 0.202 | 0.500 | 0.150 | 0.850 | -0.369 | 1.369 | a |
| Brain | Vehicle | 3 | 0.800 | 0.350 | 0.202 | 0.800 | 0.450 | 1.150 | -0.069 | 1.669 | a |
| Brain | 5HT_0.1 | 3 | 1.000 | 0.520 | 0.300 | 1.000 | 0.480 | 1.520 | -0.292 | 2.292 | a |
| Brain | 5HT_1 | 3 | 3.000 | 0.690 | 0.398 | 3.000 | 2.310 | 3.690 | 1.286 | 4.714 | b |
| Brain | 5HT_10 | 3 | 7.200 | 0.870 | 0.502 | 7.200 | 6.330 | 8.070 | 5.039 | 9.361 | c |
| HG | Control | 3 | 1.800 | 0.520 | 0.300 | 1.800 | 1.280 | 2.320 | 0.508 | 3.092 | a |
| HG | Vehicle | 3 | 2.200 | 0.870 | 0.502 | 2.200 | 1.330 | 3.070 | 0.039 | 4.361 | a |
| HG | 5HT_0.1 | 3 | 3.000 | 0.690 | 0.398 | 3.000 | 2.310 | 3.690 | 1.286 | 4.714 | ab |
| HG | 5HT_1 | 3 | 4.000 | 0.870 | 0.502 | 4.000 | 3.130 | 4.870 | 1.839 | 6.161 | bc |
| HG | 5HT_10 | 3 | 5.000 | 0.870 | 0.502 | 5.000 | 4.130 | 5.870 | 2.839 | 7.161 | c |

**Table S9.** Descriptive Statistics of Relative Expression Level of MRJP1 (norm. rp49) by Tissue and Treatment

| **Tissue** | **Treatment** | **N** | **Mean** | **SD** | **SE** | **Median** | **Min** | **Max** | **95% CI Lower** | **95% CI Upper** | **Tukey Group** |
| --- | --- | --- | --- | --- | --- | --- | --- | --- | --- | --- | --- |
| Brain | Control | 3 | 100.000 | 13.860 | 8.002 | 100.000 | 86.140 | 113.860 | 65.570 | 134.430 | b |
| Brain | Vehicle | 3 | 88.000 | 17.320 | 10.000 | 88.000 | 70.680 | 105.320 | 44.975 | 131.025 | ab |
| Brain | 5_7_DHT_0.1 | 3 | 78.000 | 13.860 | 8.002 | 78.000 | 64.140 | 91.860 | 43.570 | 112.430 | ab |
| Brain | 5_7_DHT_1 | 3 | 72.000 | 17.320 | 10.000 | 72.000 | 54.680 | 89.320 | 28.975 | 115.025 | ab |
| Brain | 5_7_DHT_10 | 3 | 45.000 | 13.860 | 8.002 | 45.000 | 31.140 | 58.860 | 10.570 | 79.430 | a |
| HG | Control | 3 | 98.000 | 17.320 | 10.000 | 98.000 | 80.680 | 115.320 | 54.975 | 141.025 | b |
| HG | Vehicle | 3 | 95.000 | 25.980 | 15.000 | 95.000 | 69.020 | 120.980 | 30.462 | 159.538 | b |
| HG | 5_7_DHT_0.1 | 3 | 62.000 | 17.320 | 10.000 | 62.000 | 44.680 | 79.320 | 18.975 | 105.025 | ab |
| HG | 5_7_DHT_1 | 3 | 48.000 | 20.780 | 11.997 | 48.000 | 27.220 | 68.780 | -3.620 | 99.620 | a |
| HG | 5_7_DHT_10 | 3 | 35.000 | 25.980 | 15.000 | 35.000 | 9.020 | 60.980 | -29.538 | 99.538 | a |
